# Optimized multi agent reinforcement learning algorithms with hybrid BiLSTM for cost efficient EV charging scheduling

**DOI:** 10.3389/frai.2025.1700664

**Published:** 2026-01-05

**Authors:** Urvashi Khekare, Rajay Vedaraj I. S.

**Affiliations:** 1 School of Mechanical Engineering, Vellore Institute of Technology, Vellore, Tamil Nadu, India; 2 School of Computer Science and Engineering, Vellore Institute of Technology, Vellore, Tamil Nadu, India

**Keywords:** MARL, EVCS, Pelican optimization algorithm, BiLSTM, decision making, charging and discharging

## Abstract

With the fast development of electric vehicles, the demand for intelligent charging management strategies in order to minimize operational costs, ensure grid stability, and enhance user satisfaction. This paper proposes a new framework that embeds multi-MARL algorithm tuned by the Pelican optimization algorithm (POA) bidirectional long short-term memory for anticipatory energy forecasting scheduling in EV charging stations—EVCS. Unlike previous works that treat forecasting, the proposed method seamlessly unifies these steps, which were hitherto considered as separate entities: optimization and then scheduling. Components within a Markov decision process formulation. The framework employs publicly available Indian Energy Exchange (IEX) day-ahead market data, where POA-tuned BiLSTM forecasts electricity price and demand with improved accuracy, feeding into the MARL controller for dynamic scheduling. Experimental results demonstrate that the proposed method reduces charging cost by 12.34%, improves state-of-charge (SOC) satisfaction by 10.25%, and increases forecasting accuracy by 8.46% compared to conventional GA, PSO, MARL, and deep learning baselines. Furthermore, simulation time is reduced by 0.456 s, confirming computational efficiency. This study presents integrated frameworks that combine POA-tuned BiLSTM forecasting with a CTDE-based MARL architecture for anticipatory EV charging scheduling.

## 1 Introduction

Electric vehicles (EVs) have emerged as an important concept in a number of nations to lower pollution levels in the environment in recent years (Shao et al., 2023a). EPRI data shows that by 2035, up to 40 and 45% of gasoline-powered vehicles will be replaced by electric vehicles in China and the United States, respectively (Pan et al., 2023). EVs are mainly utilized due to low carbon emissions and high energy efficiency, and they may even reduce the rate of climate change (Shao et al., 2023b). Moreover, due to the inherent flexibility in EV charging, auxiliary services like demand response and peak shaving can be offered to the grid (Yasmin et al., 2024). The increasing number of EVs have been accompanied by a significant increment in the number of EV charging stations (Yang et al., 2022). Charging stations serve as middlemen between EVs and electricity producers by adjusting the amount of electricity provided to EVs and the amount of electricity acquired from energy producers. Therefore, it is important to balance energy supply and load demand in EV charging stations (EVCS) (Bessa and Matos, 2012).

Intricate decision-making problems can be effectively solved with reinforcement learning (RL), which can also be used to generate intelligent EVCS recommendations Lamontagne et al. (2023). Agents can maximize long-term goals by learning from repeated trial and error in the RL method (Bachiri et al., 2023). However, standard Q-learning algorithms have difficulties in the large-scale environment with millions of EVs and thousands of charging stations (Wong et al., 2023). As the agent gets updated by receiving rewards for every positive action, penalties for unwanted actions, RL become more significant for learning and decision-making. The dimensionality is the only drawback of RL (Oroojlooy and Hajinezhad, 2023). Therefore, in recent years, MARL has been used in EV applications for optimal energy scheduling in charging stations. A collection of independent, interacting entities that share a common environment is referred to as a MULTIAGENT system (Ren et al., 2023; Ray et al., 2023). Various sectors like robotics, distributed systems, resource utilization, collective decision support systems, etc. using multi agent systems to get better results (Dong et al., 2023). It is a time-sequential issue to control the charging/discharging power for EVs to reduce charging costs while taking into consideration many unpredictable elements. As a result, the EV charging problem has been formulated as an MDP with MARL in numerous studies (Kaewdornhan et al., 2023). Deep reinforcement learning with a recurrent neural network-based EV scheduling was recommended in Li et al. (2023). It considered the uncertainties in EV users and increased the computational time, but the cost was not reduced. In Jin et al. (2023), a bi-layer steady state evaluation was done by considering the load margin index in the steady state voltage security region. Optimal load scheduling with energy management was performed using this method. Aljafari et al. (2023) suggested a multi-agent deep neural network-based energy scheduling with dynamic load change. This method also controls the charging and discharging characteristics of EV, but the computation time was high in this method. Numerous works are introduced in this field, but they still suffer from disadvantages like high computational time, high cost, etc. Therefore, this paper proposed a novel method with an MARL framework for optimal power scheduling of EVs in a charging station.

### 1.1 Research gaps and contribution

While there have been significant developments in reinforcement learning and forecasts-based energy management, the majority of studies in existing literature regarded EV charging optimization, price/load forecasting, and parameter tuning within the different studies as separate modules. This distinction frequently leads to limitations on the model’s ability to adapt, and increases dependencies on computational resources. Moreover, typical reinforcement learning approaches based on prior research have been based on static and/or reactive control strategies that do not anticipate future anticipated price/load volatility. Earlier forecasting studies were similarly limited due to their consideration toward automatic parameter tuning, therefore the forecasting accuracy, responsiveness, and convergence capability were inevitably compromised. In this study, the identified gaps in research literature of EV charging forecasting and optimization, the authors propose a new integrated hybrid framework that combines a POA tuned BiLSTM forecaster with a MARL scheduler, within a consolidated MDP formulation. The coupling of these two modules enables anticipatory and cost-driven value-added decision-making, allowing agents to adjust their charging schedules in a proactive manner using predicted IEX day-ahead prices, along with anticipated demand patterns. The hybrid POA-BiLSTM-MARL framework is a novel contribution to existing literature, attempting to make arbitraged decisions based just in time for each decision point. In summary, the significant contributions of this are work:

- A multistage reward function is developed to optimize the stability and financial performance of MARL-based EV charging decisions.
- A highly efficient BiLSTM-based forecasting model was developed to accurately forecast the short-term electricity price and load.
- The POA is used to automatically tune the hyper-parameters of the BiLSTM, which results in the improvement of forecast accuracy by up to 8.46%.
- The resulting hybrid framework reduces charging cost by 12.34%, improves SOC satisfaction by 10.25%, and runtime by 0.456 s compared to the state-of-the-art baselines.

In contrast to prior research which addressed reinforcement learning and forecasting individually, this work presents a hybrid framework that integrates forecasting, meta-optimization, and multi-agent control into one decision-making framework. The Pelican optimization algorithm (POA) is not just utilized as a standalone optimizer but is instead automatically tuning the BiLSTM model’s hyperparameters to facilitate enhanced forecasting reliability. These forecasts are then optimally incorporated in the MARL environment for anticipatory scheduling decisions. Additionally, CTDE-based MARL structure promotes scalable and cooperative learning while increasing convergence stability and reducing compute time across multiple EV agents. The design is uniquely integrated into the decisions as a methodological advancement from prior works that treat them separately.

The remainder of this paper is organised as follows: Section 2 discusses some of the existing works related to MARL in EV applications. Section 3 briefly explains the proposed methodology with its techniques. Results and discussion of the proposed work with comparative results are presented in Section 4. Conclusion of the proposed work and future scope are given in Section 5.

## 2 Related works

Some of the existing works related to these fields are discussed in this section.

In order to address the scalability challenges of large-scale smart grid systems, Alqahtani et al. (2022) suggested reformulating a mixed-integer programming (MIP) model into a decentralized Markov decision process (DEC-MDP) model and solving it using a MARL algorithm. MIP method schedules the energy and feeds it to a specific location in each step. The MIP decision model is then reformulated as a DEC-MDP, in which the EVs were first arranged in a centralized manner and subsequently implemented in a decentralized manner.

Qiu et al. (2022) recommended a novel method to enhance the performance of the EV scheduling process. MARL-based method was used for aligning the EV power in a discrete and continuous process. The performance of this method was enhanced by including a decentralized partially observable MDP integrated with a hybrid MARL method Yang et al. (2020). The proximal policy optimization (PPO) algorithm was employed to calculate the *Q*-value in the network. This method optimally reduced the load shedding and enhanced the stability of the distribution system.

Mishra and Singh (2025) suggested a DRL method in an EVCSs in order to minimize the expenses in the charging station. A user equilibrium traffic assignment problem (UE-TAP) was used in this method to design the power and transportation system. In addition to reducing the scope of interactions between agents, neighbourhood factorization implicitly maintains the global information within a pair. The learning function was approximated by applying the mean-field theory in the suggested method. This method optimally reduced the charging cost in the charging station, which was an advantage for the EV consumers.

Xu et al. (2020) recommended a neural network-based Q-learning algorithm for home energy management. Extreme learning machine and Q-learning were suggested under a data-driven framework to increase the computational frequency in this method. Home appliances and EV charging stations were considered as a load, a suggested method to satisfy the load demand optimally Alamir et al. (2023). The decision-making procedure for accurate and realistic scheduling was done by the suggested feedforward NN. This method reduced the electricity bill and satisfied the demand response of the consumer optimally.

Li et al. (2022) recommended a novel method to reduce the losses in the distribution transformer in an EV charging station. An LSTM-based NN method was included in this work to evaluate the uncertainties caused by the load demand. The EV charging station’s charging issue was rectified by integrating a multi-agent deep reinforcement learning method. The recommended method opponent network processes to process the EVs’ information by using the attention mechanism. This method effectively directs the actor network’s creation of coordinated strategies. Each agent’s decision-making capabilities were built through offline training and then implemented online to choose the control actions according to the most recent information about the condition of the system. Recent research has also focused on extending reinforcement learning to sustainability-oriented EV operations. Zhou et al. (2025) introduced a multi-agent DRL framework for self-consumption scheduling in highway EV charging stations, while Satpathy et al. (2025) explored sustainable and technology-driven strategies for enhanced EV performance and integration. These studies reflect the growing shift toward renewable-aware and market-adaptive EV scheduling models.

Unlike Qiu et al. (2022), Mishra and Singh (2025), Xu et al. (2020), Li et al. (2022), Jamjuntr et al. (2024), Zhou et al. (2025), Satpathy et al. (2025), and Alduailij (2025), which combine MARL with conventional forecasting or optimization, the proposed work uniquely integrates POA-tuned BiLSTM within a CTDE-based MARL framework. This addresses both forecasting uncertainty and hyperparameter sensitivity simultaneously, which has not been jointly studied in recent literature. Table 1 provides a detailed comparative summary of recent MARL-based EV scheduling studies, highlighting the distinct methodological advances, datasets, and performance benchmarks. The proposed framework distinguishes itself by integrating a POA-tuned BiLSTM forecaster within a CTDE-based MARL structure, bridging the forecasting and optimization gap identified in prior research.

**Table 1:** Comparative literature review and novelty summary.

References	Methodology/core approach	Forecasting or optimization component	Dataset or case study	Reported performance/key outcomes	Limitations/gaps identified	Novelty difference from proposed work
Qiu et al. (2022)	Hybrid MARL for EV resilience control	Deep RL-based scheduling without explicit forecasting	IEEE Test Systems	Improved resilience and load balancing	No integration of prediction or meta-optimization	Proposed model unifies MARL with predictive POA-tuned BiLSTM forecasting
Mishra and Singh (2025)	Multi-agent deep RL for EVCS game model	None (static pricing)	Synthetic EVCS dataset	Reduced cost and congestion	Ignores future demand and price variability	Proposed work introduces anticipatory control through POA-BiLSTM
Xu et al. (2020)	Multi-agent Q-learning for home energy management	None; used fixed tariffs	Smart home simulation	7–9% cost saving	Not scalable to multi-EV environments	Current work extends to large-scale EVCS with dynamic market pricing
Li et al. (2022)	LSTM-aided MARL for transformer lifetime optimization	LSTM forecasting (manual tuning)	Grid-connected EV network	Improved transformer health index by 5%	Hyperparameters manually fixed; no meta-optimization	POA automates hyperparameter tuning for BiLSTM forecasting
Jamjuntr et al. (2024)	Adaptive MARL for EV networks in Thailand	Rule-based price input	Regional testbeds	Reduced cost by 8.2%	Lacks learning-based forecasting	Proposed framework embeds predictive forecasting with MARL
Ren et al. (2023)	Dynamic power allocation for fast charging	None	Extreme-fast EVCS	Minimized overload risk	No learning or multi-agent coordination	Present study integrates multi-agent control under CTDE strategy
Aljafari et al. (2023)	Deep neural network for dynamic pricing scheduling	Feed-forward NN	Simulated grid	Enhanced flexibility in charging/discharging	No reinforcement learning integration	Current model fuses deep learning with MARL and POA meta-optimization
Kamrani et al. (2025)	Multi-agent DRL for fair EV dispatch	PPO-based coordination	IEEE 33-bus system	Stable convergence under dynamic load	Forecasting not included	Proposed system embeds price-demand forecasting into MARL state
Shojaeighadikolaei et al. (2024)	Centralized vs. decentralized MARL for EV charging	None	Simulation study	Improved control efficiency	No integration of forecasting or optimization	Proposed CTDE + POA + BiLSTM hybrid bridges both aspects
This work	POA-tuned BiLSTM + MARL under CTDE	BiLSTM forecasting optimized by POA	IEX Day-Ahead Market (India)	Cost ↓ 12.34%, SOC ↑ 10.25%, Forecast Accuracy ↑ 8.46%	—	Integrates forecasting, meta-optimization, and multi-agent scheduling in a unified MDP framework

## 3 Proposed methodology

EVs are the foundation of future mobility due to their advantages, like no emissions and being eco-friendly. EVs can also be blended as distributed energy resources (DERs) into the smart grid by using a vehicle-to-grid (V2G) scheme. Renewable energy sources are mostly used a distribution generation in modern days due to their advantages like low carbon emissions and pollution-free. In this work, a solar photovoltaic system (PV) is used as a source for the EVCSs. Excess power generated from the PV sources is stored in the battery energy storage system for future use. This paper proposes a novel framework for EV energy management scheduling based on reinforcement learning in achieving an efficient EVCSs-based BiLSTM to satisfy demand response. A MARL method schedules the energy intensity of an EVCSs to control the maximum performance level of the grid. BiLSTM reduces the inaccuracies of autonomous predictions of energy calculations with the help of EV agents. The energy scheduling charging issue is reduced using the proposed MARL. After completing the training process, all of the agents are trained centrally to create coordinated control strategies and make decisions based on local inputs. BiLSTM is used in this work to enable the EV charging station to make reasonable decisions concerning historical decision information. The POA is used in this work to optimize the hyperparameters of the BiLSTM.

### 3.1 Modelling of EV

EVs have gained a lot of interest due to cost-effective and environmentally friendly alternative for EV with internal combustion engines. Because they reduce reliance on fossil fuels and greenhouse gas emissions, EVs are desired. It takes equipment to charge EVs, which is essential for their daily use and grid integration (Jang et al., 2020). Batteries’ capacity to charge and discharge is used to model EVs. The majority of batteries used in EVs are lithium-ion batteries because of their energy density and durability. The cells of these batteries are arranged in a module by connecting them in series and parallel. The components of a charging station usually include a power outlet, EV connector, attachment plug, charge cord, charge stand, and protective system. The main element influencing charging time, cost, equipment, and grid impact is the charger power level. A lot of things need to be considered when building the charging station are as follows;

$E_{i}^{\mathrm{nom}}$ (kWh) is nominal battery capacity of EV *i*, (Trojovský and Dehghani, 2022). Power balance in EVCS is represented in Equation (1) as follows:


$$\;P_{\text{grid},t}+P_{\mathrm{PV},t}+\sum_{i}p_{i,t}^{\mathrm{dis}}=P_{\mathrm{aux},t}+\sum_{i}p_{i,t}^{\mathrm{ch}}$$ (1)

where $P_{\text{grid},t}>0$ , means import, $p^{\mathrm{dis}}$ positive means EV discharging, *t* is the time step (h), $P_{\text{grid},t}$ , represent net real power at grid intertie (>0 import, <0 export), $P_{\mathrm{PV},t}$ represent PV real power available at bus, $p_{i,t}^{\mathrm{dis}}$ is the discharging power set-point for EV *i,* $p_{i,t}^{\mathrm{ch}}$ is charging power set-point for EV *i*, $P_{\mathrm{aux},t}$ station auxiliary load (HVAC, lighting). Load demand in the EVCS is based on the requirement for EV arrival. All power values are in (kW). The energy requirement from the target SOC Equation (2) given below,


$$E_{i,\mathrm{req}}=ed_{i}$$ (2)

where $E_{i,\mathrm{req}}$ , represents the energy (kWh) required to reach the target SOC for EV *i*, e is energy consumption per km (kWh/km), $d_{i}$ is the trip distance (km). Power is given by Equation (3)


$${\bar{p}}_{i}=\frac{E_{i,\mathrm{req}}}{T_{i}}$$ (3)

with $T_{i}$ = available dwell time (h). $E_{i}^{\mathrm{nom}}$ represents nominal battery energy capacity (kWh) of EV *i*, $\mathrm{SO}C_{i}$ represents the SOC of EV *i*. The limit of charging and discharging of EVs is considered based on the SOC of EVs. This SOC can be evaluated using below Equation (4):


$$\mathrm{SO}C_{i,t+1}=\mathrm{SO}C_{i,t}+\frac{\eta_{\mathrm{ch}}.p_{i,t}^{\mathrm{ch}}-\frac{1}{\eta_{\mathrm{dis}}}p_{i,\;t}^{\mathrm{dis}}}{E_{i}^{\mathrm{nom}}}\mathrm{\Delta t}$$ (4)

where $p_{i,t}^{\mathrm{ch}}$ is charging power (kW), $p_{i,\;\;t}^{\mathrm{dis}}$ is discharging power (kW), Δta is time step (h), $E_{i}^{\mathrm{nom}}$ is capacity (kWh), $\eta_{\mathrm{ch}}$ & $\eta_{\mathrm{dis}}$ are charging and discharging efficiencies.

The limit of the EV battery (Kumar et al., 2023) is set based on below Equation (5) for all *t*;


$$\mathrm{SO}C_{i}^{\min}\leq \mathrm{SO}C_{i.t}\leq \mathrm{SO}C_{i}^{\max}$$ (5)

where, $\mathrm{SO}C_{i}^{\min}$ & $\mathrm{SO}C_{i}^{\max}$ are allowed SOC bounds. Cost for interval *t* is given by Equation (6):


$$\;C_{t}=\lambda_{t}\;P_{\text{grid},t}^{\mathrm{imp}}\;\mathrm{\Delta t}-\lambda_{t}^{\exp}\;P_{\text{grid},t}^{\exp}\;\mathrm{\Delta t}$$ (6)

where,


$$\;P_{\text{grid},t}^{\mathrm{imp}}=\max(P_{\text{grid},t},0),\;\;P_{\text{grid},t}^{\exp}=\max(-P_{\text{grid},t},0)$$

Here $P_{\text{grid},t}$ is the net power exchange with the grid at time t (kW), positive for imports and negative for exports. $P_{\text{grid},t}^{\mathrm{imp}}$ and $P_{\text{grid},t}^{\exp}$ are the imported and exported power magnitudes, respectively. $\lambda_{t}$ is the import tariff (INR/kWh), $\lambda_{t}^{\exp}$ is the feed-in tariff, and Δt is the time-step length (h). This convention ensures that $C_{t}>0\;$ during net imports (a cost) and $C_{t}<0\;$ during net exports (a revenue or saving).

### 3.2 MARL

**Figure 1:**
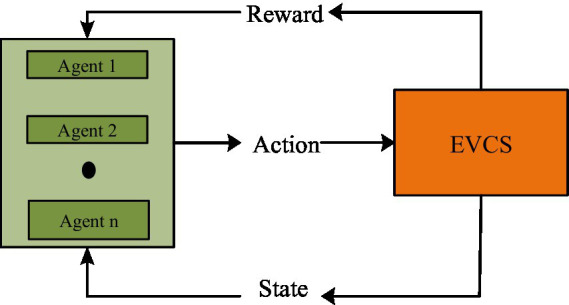
Multi-agent reinforcement learning.

MARL addresses sequential decision-making problems, but with more than one agent involved. A group of independent agents interacts with their surroundings in MARL to figure out how to accomplish their goals. Although MDPs are useful in simulating optimal decision-making in stochastic single-agent systems, a different representation is needed for multi-agent environments. The fundamental stationary assumption of an MDP is broken when all agents act together, altering the state dynamics and expected rewards. MDPs may appear to the agent fully or partially. The way in which agents interact, cooperative, competitive, or mixed, and whether they operate concurrently or sequentially, determines how the problem is represented in a multi-agent context. For high-dimensional situations, MARL performs better than deep reinforcement learning and other optimization methods (Canese et al., 2021). It can execute in a decentralized manner as well as conduct training in a centralized manner, which reduces execution time and places fewer restrictions on the agents. Because large-scale energy problems involve many factors that are dynamic in nature and call for quicker decision-making processes, MARL is an effective option for handling them. Additionally, a multistep reward function is suggested in place of an immediate reward function, taking into account how suitable shortened steps can enhance the MARL-based approaches’ economic performance and learning speed in the power market. Figure 1 represents the MARL in the proposed work.

The MDP is a powerful modelling technique for sequential decision-making issues (Shao et al., 2023b), acting as a crucial connection between reinforcement learning algorithms and optimization problems with unpredictable state transitions (Zhang et al., 2021). The important functions of MDP are as follows:

*State*: The charging demand restrictions and aim of the optimization problem are discretized over a time scale to formulate the state based on the SOC of EVs.

*Action*: Consistency and relevance are ensured by the development of actions in accordance with decision variables.

*Reward function*: This takes into consideration the charging station’s power limitations as well as the optimization goal. The reward function incorporates the charging station’s power. This is because it is impossible to impose the charging station’s overall power limit as a constraint for each charging pile due to the local and decentralized nature of the charging and discharging decisions made for each charging pile (Jain et al., 2022). The local state of each agent is expressed in Equations (7) and (8) as follows:


$$\zeta_{i}=\{N_{\mathrm{csj}}^{i},L_{\mathrm{csj}}^{i},a_{i}\}$$ (7)

$$\zeta_{i}=[\begin{array}{ccccccc} N_{\mathrm{cs}0}^{0} & \cdots & N_{\mathrm{cs}3}^{0} & L_{\mathrm{cs}0}^{0} & \cdots & L_{\mathrm{cs}3}^{0} & a_{0}\\ N_{\mathrm{cs}0}^{1} & \cdots & N_{\mathrm{cs}3}^{1} & L_{\mathrm{cs}0}^{1} & \cdots & L_{\mathrm{cs}3}^{1} & a_{1}\\ \vdots & \ddots & \vdots & \vdots & \ddots & \vdots & \vdots \\ N_{\mathrm{cs}0}^{k-1} & \cdots & N_{\mathrm{cs}3}^{k-1} & L_{\mathrm{cs}0}^{k-1} & \cdots & L_{\mathrm{cs}3}^{k-1} & a_{k-1} \end{array}]$$ (8)

where, $\zeta_{i}$ indicates each agent, $a_{i}$ denotes the previous action of the agent, $L_{\mathrm{cj}}^{i}$ means the number of EVs available currently in the charging station with neighbouring agents, k signifies the number of other agents, and represents the length of the queue in the charging station. Each agent can record the current count of charging EVs, the current length of the charging station’s queue, and the neighbouring agent’s current action plan. The agent considers how many vehicles are charging in the EVCS within its control range, as well as how far the EVCS has to go.

### 3.3 BiLSTM

Time-series forecasting of electricity prices and EV demand requires a model capable of capturing both short- and long-term dependencies. In this work, a BiLSTM network is adopted because it processes sequences bidirectionally, which allows the model to exploit contextual data from the timeline simultaneously Suebsombut et al.(2021). Unlike a standard LSTM, which only propagates information forward, the BiLSTM improves learning of temporal correlations that are critical for volatile market prices (Gen and Lin, 2023). In the proposed framework, the BiLSTM is trained on historical IEX data to predict 15-min-ahead price signals. These predictions are then supplied as inputs to the MARL scheduler. By explicitly incorporating forecasting into the scheduling loop, the agents are able to anticipate tariff fluctuations rather than reacting to them (Houran et al., 2023). The hyperparameters of the BiLSTM, including hidden layer size, learning rate, and dropout ratio, are optimized automatically using the Pelican optimization algorithm.

**Figure 2:**
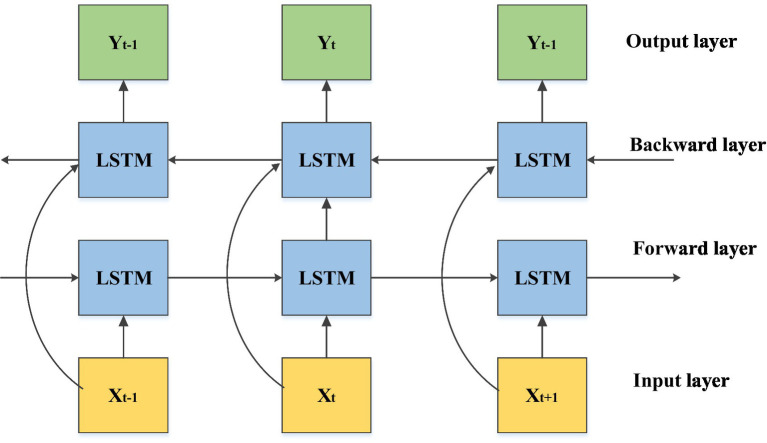
BiLSTM model.

Figure 2 shows the BiLSTM model. To analyse various EVs’ charging or discharging, the predicted data is provided as an input to the MARL-based proposed approach. In the proposed framework, the POA is employed solely to optimize the hyperparameters of the BiLSTM forecasting model. These include the number of hidden layers, neuron count, learning rate, dropout ratio, and look-back window size. This optimisation enhances forecasting accuracy for short-term electricity price and demand, which subsequently improves the quality of decisions made by the MARL scheduler. The MARL learning parameters (*α*, *γ*, *ε*) remain fixed throughout training and are not influenced by POA.

### 3.4 POA

POA is a bio-inspired algorithm technique that imitates the hunting behaviour of pelicans. For finding out the best value in the global optimal solution, this approach offers the best exploration and exploitation (Pande and Khekare, 2024). POA method is initialized based on below Equation (9):


$$X={\left[\begin{array}{c} X_{1}\\ \vdots \\ X_{i}\\ \vdots \\ X_{N} \end{array}\right]}_{N\times m}={\left[\begin{array}{ccccc} X_{1,1} & \cdots & X_{1,j} & \cdots & X_{1,m}\\ \vdots & \ddots & \vdots & ⋰ & \vdots \\ X_{i,1} & \cdots & X_{i,1} & \cdots & X_{i,m}\\ \vdots & ⋰ & \vdots & \ddots & \vdots \\ X_{N,i} & \cdots & X_{N,j} & \cdots & X_{N,m} \end{array}\right]}_{N\times m}$$ (9)

where, X represent the population matrix and $X_{i}$ represents *i*^th^ pelican. A possible value is denoted by rows, and the column shows an optimal value. The population initialization takes place, based on lower and upper bounds as given in Equation (10)


$$\begin{array}{l} X_{i,j}=A_{j}+R\cdot (B_{j}-A_{j})\\ i=1,2,\ldots \ldots ,N\\ j=1,2,\ldots \ldots ,m \end{array}\}$$ (10)

where, $X_{i,j}$ represent the *j*^th^ variable value in *i*^th^ candidate solution, m is the problem variable, R , N , B , A , represents the random population range, the total number of the population, the upper bound, and the lower bound in the search range, respectively. The fitness function is evaluated using Equations (11) and (12) as follows:


$$F={\left[\begin{array}{c} F_{1}\\ \vdots \\ F_{i}\\ \vdots \\ F_{N} \end{array}\right]}_{N\times 1}={\left[\begin{array}{c} F(X_{1})\\ \vdots \\ F(X_{i})\\ \vdots \\ F(X_{N}) \end{array}\right]}_{N\times 1}$$ (11)

F=min(cost) (12)

where F is the fitness function. The selection of weight parameters to achieve optimal power flow having minimum cost is the objective function.

#### 3.4.1 Phase 1 (exploration)

Within the search space, the position of POA is randomly generated. POA can accurately explore the problem-solving space with Equations (13) and (14):


$$X_{i}^{P1}=\{\begin{array}{ll} X_{i,j}+R\cdot (P_{j}-R_{1}\cdot X_{i,j}), & F_{p}<F_{i}\\ X_{i,j}+R\cdot (X_{i,j}-P_{j}), & \text{else} \end{array}$$ (13)

$$X_{i}=\{\begin{array}{ll} X_{i}^{P1}, & F_{i}^{P1}\\ X_{i} & \text{else}. \end{array}$$ (14)

where, $X_{i}^{P1}$ , $P_{j}$ , $F_{p}$ , $F_{i}^{P1}$ , represent the exploration phase status, the location of prey in *j*^th^ dimension, the objective function value and the fitness function, respectively, based on phase 2.

#### 3.4.2 Phase 2 (exploitation)

**Figure 3:**
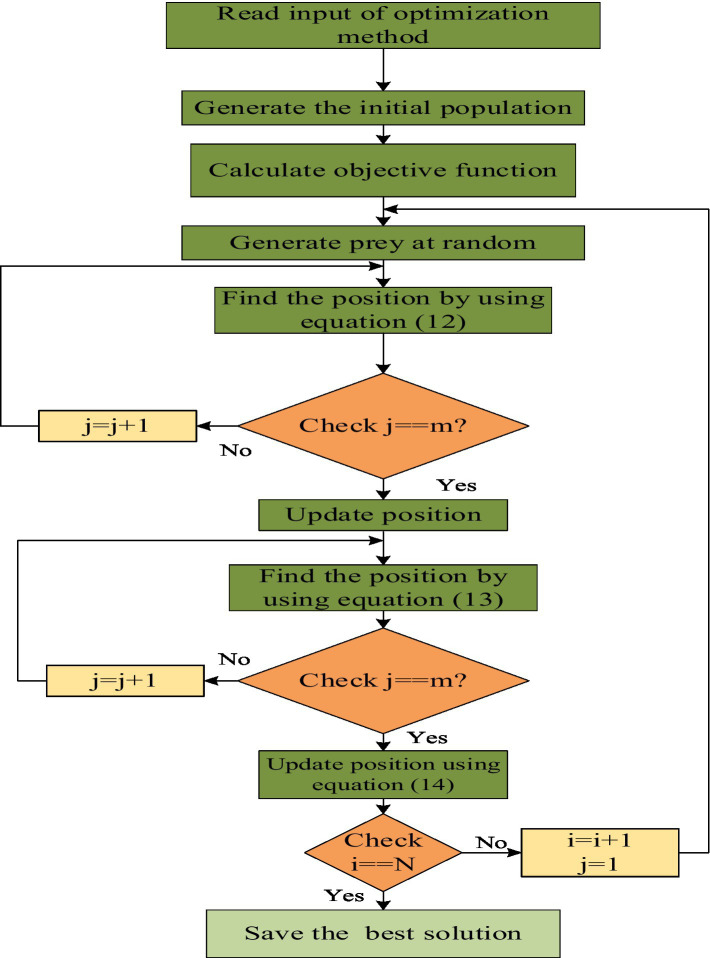
Flowchart for POA.

The hunting behaviour of pelicans is used for this section. This section is designed as follows: The position update phase takes place and updates the parameters for the next iterations with the following Equations (15) and (16):


$$X_{i,j}^{P2}=X_{i,j}+R(1-\frac{t}{T})\cdot X_{i,j}\cdot (2R-1)$$ (15)

$$X_{i}=\{\begin{array}{ll} X_{i}^{P2}, & F_{i}^{P2}\\ X_{i} & \text{else}. \end{array}$$ (16)

where, $X_{i,j}^{P_{2}}$ , t , T , $F_{i}^{P2}$ , indicates a new position based on phase 2, the iteration counter, a maximum number of iterations and an updated objective function, respectively. This method will choose the optimized weight parameters with minimum cost. Figure 3 shows the flow diagram of the proposed POA method. Algorithm 1 represents the steps included in tuned BiLSTM with CTDE-MARL EV scheduling.

ALGORITHM 1 POA-tuned BiLSTM → CTDE-MARL EV scheduling

1. Preprocess data:
   1.1 Normalize D_market; engineer features (price_lag_k, volume_lag_k, time_of_day, weekday, PV_forecast, temperature).
   1.2 Split D_market into train/val/test
2. POA hyperparameter tuning for BiLSTM:
   2.1 Initialize POA population P (each particle encodes BiLSTM hyperparams: num_layers, hidden_units, learning_rate, dropout, lookback_window).
   2.2 For iter = 1…max_iters_POA:
        For each particle p in P:
             - Build BiLSTM model M_p with hyperparams(p).
             - Train M_p on D_market_train for N_epoch (early stopping on val loss).
          - Evaluate val_loss_p
        - Update P according to POA update rules (exploration/exploitation).
   2.3 Select best particle p* → final hyperparams_h*.
3. Train final BiLSTM forecaster F_forecast with hyperparams_h* on combined train+val data.
4.  Apply POA only to tune BiLSTM hyperparameters (network depth, learning rate, temporal window size).
   4.1 Train BiLSTM with optimised parameters.
   4.2 Update MARL policies using PPO with fixed α, γ, and ε values.
   4.3  Prevent any POA interaction with MARL policy learning.
5. MARL training (CTDE):
   5.1 Initialize centralized critic network Q_c and decentralized actor networks {π_i}.
   5.2 For episode = 1…episodes:
         Reset environment E_aug with sampled EV arrival/departure traces from D_ev.
         For step = 1…max_steps:
              - For each agent i: observe s_t^i and select a_t^i ~ π_i(s_t^i).
              - Execute joint action a_t = {a_t^i}; environment returns s_{t+1}, r_t, done.
              - Store transitions (s_t, a_t, r_t, s_{t+1}) in centralized replay buffer.
         - After K steps: update Q_c and π_i parameters using CTDE update rules
         - Periodically update target networks and evaluate on validation traces.
6. Evaluate:
   - Evaluate trained policies on test traces. Report: cost reduction, SOC reliability, forecasting RMSE/MAE/MAPE/R², computation time.
End.

### 3.5 Dataset description

The forecasting component in this study relies on publicly available day-ahead market (DAM) price data obtained from the Indian Energy Exchange (2025). For longer-term historical coverage, a curated Kaggle mirror dataset (Mukund, 2024) is also utilized. To ensure transparency and reproducibility, the forecasting component in this study relies on publicly available day-ahead market (DAM) price data obtained from the Indian Energy Exchange (IEX). The IEX publishes 15-min interval price data from April 1st, 2022, onwards. In order to achieve longer-term historical coverage, the paper leverages a curated Kaggle mirror dataset ranging from 2019 to 2024. Each record includes approximately 35,040 records per year (96 entries per day) for variables of interest, such as electricity price in INR/kWh, time block, and market-clearing price. Temporal splitting was carried out to preserve the chronological integrity of the dataset for experimental evaluation. Thus, the training set consists of data from 2019 to 2022, the validation set consists of the full year of 2023, and the independent test set ranges from January to June 2024. This ensures that model evaluation is performed on unseen future data and follows the requirements of realistic forecasting scenarios.

### 3.6 Markov decision process formulation

For rigorous validation of the proposed method, it is necessary to establish a well-defined mathematical framework. This section outlines the exact MDP structure, state and action spaces, reward design, and data-driven forecasting module. The consolidation of these components enhances reproducibility and eliminates interpretational uncertainty.

#### 3.6.1 The EV charging scheduling is represented as a finite-horizon MDP

Equation (17) represents the EV charging scheduling as a finite-horizon MDP


M=S,A,P,R,γ (17)

- State space (S): At time t(h) , the system state is given in Equation (18)

$$s_{t}=\{\mathrm{SO}C_{i}^{t},P_{\text{grid}}^{t},P_{\mathrm{PV}}^{t},\lambda_{t},Q_{t}L_{t}\}$$ (18)

where $\mathrm{SO}C_{i}^{t}$ is the state-of-charge of EV *i*, $\;P_{\text{grid}}^{t}\;$ is the available grid power (kW), $P_{\mathrm{PV}}^{t}$ is photovoltaic generation, $\lambda_{t}$ is the market price (INR/kWh), and $Q_{t}$ denotes, queue, and $L_{t}$ is transformer loading.

- Action space (A): Each agent *i* selects an action $a_{i}^{t}\in [0,P_{i}^{\max}]\;a$ , representing the charging/discharging rate in kW, bounded by charger capacity and SOC limits.
- Transition probability (P): Defines the stochastic evolution of SOC and system load, influenced by EV arrivals, departures, and renewable generation uncertainty.
- Reward function (R): To ensure commensurate scaling across economic and technical objectives, we use unit-free terms, Equation (19) represents Reward function as follows:

$$\begin{array}{l} R_{t}=-\lambda_{c}\;\frac{C_{t}}{C_{\max}}-\lambda_{p}\;\frac{P_{t}^{\text{peak}}}{P_{\mathrm{trf}}^{\text{rated}}}-\lambda_{\ell }\;\max(0,\frac{L_{t}}{L_{\text{limit}}}-1)\\ -\lambda_{\mathrm{SOC}}\;\frac{1}{N_{\mathrm{EV}}}\sum_{i=1}^{N_{\mathrm{EV}}}|\mathrm{SO}C_{i,t}-\mathrm{SO}C_{i}^{\text{target}}| \end{array}$$ (19)

where $C_{t}$ is the grid energy cost at time t , $C_{\max}$ is a reference maximum cost (e.g., maximum daily cost under naive charging), $P_{t}^{\text{peak}}$ is station power at time t , $\frac{P_{t}^{\text{peak}}}{P_{\mathrm{trf}}^{\text{rated}}}\;$ is transformer rated power, $L_{t}$ is transformer loading, $L_{\text{limit}}$ is its allowable limit, $\mathrm{SO}C_{i,t}$ and $\mathrm{SO}C_{i}^{\text{target}}$ are actual and target SOC for EV i , $N_{\mathrm{EV}}$ is the number of EVs, $\lambda_{c},\lambda_{p},\lambda_{\ell },\lambda_{\mathrm{SOC}}\geq 0\;$ control the trade-off between cost, peak demand, grid safety, and SOC satisfaction. This normalization makes each term lie in [0,1] under typical operation and prevents unit-driven domination.

- Discount factor (*γ*): Set within [0.9, 0.99] to balance short-term savings and long-term stability.

#### 3.6.2 Agent coordination and training protocol

A centralized training, decentralized execution (CTDE) strategy is adopted: During training, a global critic has access to all states for stable gradient updates. During execution, each EV agent makes decisions using only local SOC and queue information. Proximal policy optimization (PPO) is employed for policy learning with shared parameters across homogeneous agents.

#### 3.6.3 Forecasting and data integration

Forecasted $\lambda_{t}$ and baseline demand from the POA-tuned BiLSTM are injected into the state vector, enabling anticipatory scheduling. Training episodes are defined as 24-h horizons, with each step representing 15-min intervals.

#### 3.6.4 Mathematical integration of forecasting and MARL

The proposed POA-BiLSTM-MARL framework integrates price and demand forecasting with multi-agent reinforcement learning within a unified Markov decision process. The BiLSTM module provides short-term predictions of electricity price and system demand, which are embedded directly into the decision-making cycle of the MARL agents, enabling proactive and cost-aware charging strategies.

- State representation

At each time step t , the state observed by EV agent i is defined as Equation (20):


$$S_{t}^{i}=[\mathrm{SO}C_{i,t},{\hat{\lambda }}_{t},{\hat{L}}_{t},P_{\mathrm{PV},\;t},Q_{t}]$$ (20)

where $\mathrm{SO}C_{t}^{i}$ denotes the current state of charge of EV i , ${\hat{\lambda }}_{t}$ , and ${\hat{L}}_{t}$ are the forecasted electricity price and demand from the BiLSTM model, $P_{\mathrm{PV},\;\;t}$ is available photovoltaic power, and $Q_{t}$ is the queue length at the charging station. This formulation allows agents to anticipate both market conditions and infrastructure constraints when selecting actions.

- Action policy

Each agent determines its charging or discharging decision using a parameterized policy network represented in Equation (21):


$$a_{t}^{i}=\pi_{\theta }(S_{t}^{i})$$ (21)

where $\pi_{\theta }$ is the policy function optimized through PPO under a centralized training and decentralized execution (CTDE) scheme.

- Reward structure

$$r_{t}^{\left(i\right)}=R_{t},\forall i$$ (22)

This unified reward Equation (22) combines normalized grid energy cost, peak demand stress, transformer overloading risk, and deviation from target SOC. The energy cost term explicitly incorporates the forecasted electricity price through the revised cost formulation (Equation 6), ensuring that scheduling decisions are economically guided by the BiLSTM predictions. The reward feedback is used to update the centralized critic and decentralized actor policies. Furthermore, the POA dynamically tunes the PPO learning parameters α , γ , and ∈ by maximizing cumulative reward over training episodes. This creates a closed-loop interaction where improved forecast accuracy contributes to better reward optimization, which in turn enhances charging performance and grid stability. This integrated structure establishes a coherent link between forecasting accuracy and real-time scheduling efficiency, ensuring consistency across the MDP formulation, reward modelling, and agent learning processes.

**Figure 4:**
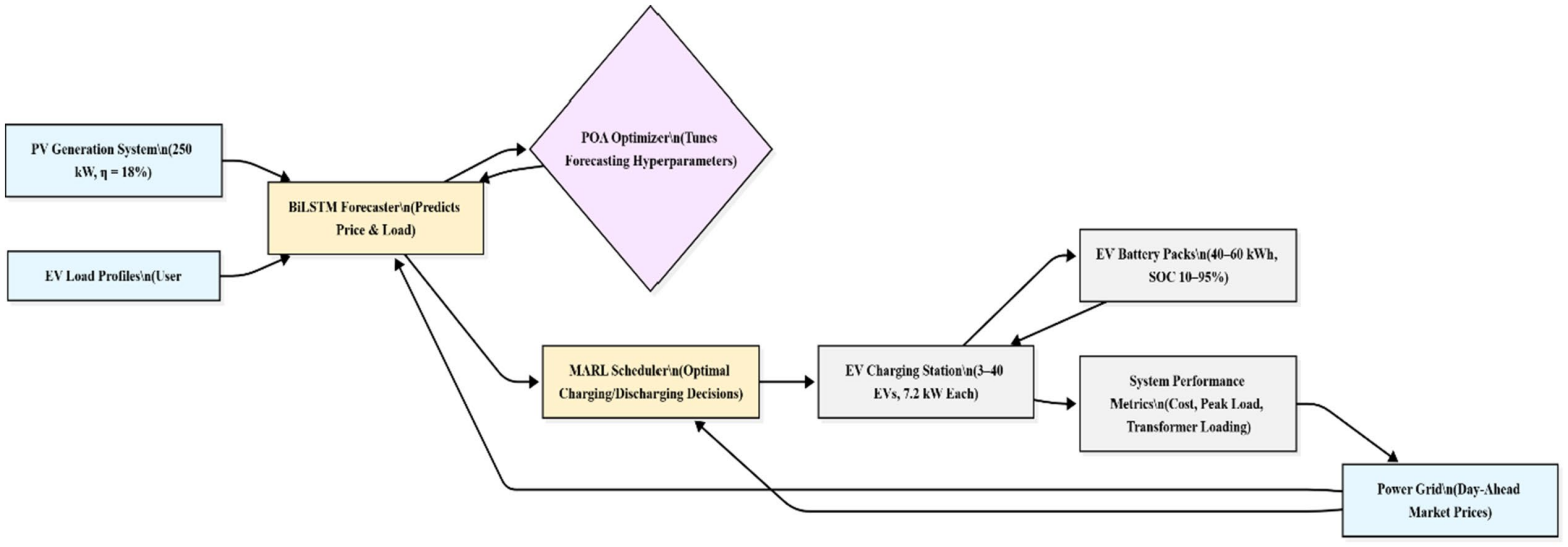
Detailed model system of the study.

Figure 4 shows a detailed model system of the study, showing the interaction among the PV generation, BiLSTM forecaster, POA optimiser, MARL scheduler, and EV charging infrastructure. The BiLSTM forecaster predicts the short-term variability of electricity price and demand profiles based on PV and grid data, while the POA optimiser tunes the parameters accordingly. Multiple EV agents, i.e., EV1, EV2, EV3, are included in the MARL environment modeled as independent learners possessing their individual SOC, battery capacity, and arrival/departure characteristics.

### 3.7 Experimental scenario description

The simulation framework models an EV charging station with PV generation, multiple charging piles, and interconnection to the grid. For instance, three representative EV agents, EV1, EV2, and EV3, are explicitly modeled in the MARL environment. Each EV corresponds to one charging pile in the station, and is viewed as an independent agent that is characterized by its own battery capacity, SOC, and dwell time characteristics. Specifically, EV1, EV2, and EV3 are initialized with SOCs of 40, 55, and 70% and nominal capacities of 40 kWh, 50 kWh, and 60 kWh, respectively. These variations capture the heterogeneity of real users while having homogeneous charger specifications: 7.2 kW AC Level-2. This will later be extended to 40 EVs for large-scale evaluation.

EVs arrive according to a stochastic Poisson process, with an average inter-arrival time of 20 min during peak hours and 45 min during off-peak hours. Dwell times are uniformly distributed between 1.5–4 h. Each EV has a battery capacity in the range of 40–60 kWh, charged by 7.2 kW AC Level-2 chargers, V2G capable wherever applicable. SOC limits are between 10 and 95%. Pricing follows the IEX day-ahead market price with a resolution of 15 min. Network constraints include a 500 kW transformer limit and feeder capacity as per the local distribution norm. This explicit scenario description will ensure physical consistency and reproducibility across all reported experiments.

Table 2 summarizes the hyperparameter settings and convergence criteria adopted for all comparative algorithms, including GA, PSO, MARL, LSTM, BiLSTM, and the proposed POA-BiLSTM-MARL framework. Each method was trained and executed under identical datasets, runtime budgets, and computational conditions to ensure a fair and reproducible comparison.

**Table 2:** Hyperparameter configurations and convergence criteria for benchmark algorithms.

Algorithm	Key hyperparameters	Convergence/stop criterion
GA	Pop. = 50, Crossover = 0.8, Mutation = 0.05	Δ Fitness < 1 × 10^−4^ or 100 iters
PSO	Swarm = 50, *w* = 0.7, *c*₁ = *c*₂ = 1.5	Δ Global best < 1 × 10^−4^ (5 iters)
MARL (PPO)	lr = 0.0003, *γ* = 0.95, Batch = 256, Clip = 0.2	Δ Reward < 1% or 200 episodes
LSTM	2 layers × 128 units, lr = 0.001, Batch = 64	Early stopping (15 epochs)
BiLSTM	Same as LSTM	Early stopping (15 epochs)
POA-BiLSTM-MARL	POA pop = 30, iter = 50; *α*, *γ*, *ε* adaptive	Δ Reward < 1 × 10^−3^ (10 episodes)

The GA and PSO algorithms employed typical evolutionary parameters with fixed population or swarm sizes and terminated when the improvement in the objective value became negligible or when the maximum iteration count was reached. For reinforcement learning (MARL using PPO), convergence was defined as the point where the average episode reward stabilized with a variation of less than 1% over 10 evaluation episodes. The deep learning forecasters (LSTM and BiLSTM) were trained using the Adam optimizer with a learning rate of 0.001 and an early stopping mechanism triggered after 15 epochs of no improvement in validation loss. The proposed POA-BiLSTM-MARL model utilized the Pelican optimization algorithm to adaptively tune the BiLSTM and MARL hyperparameters, achieving automatic convergence when cumulative reward improvement was below 10^−3^ for 10 consecutive episodes. These consistent parameter settings and stopping conditions guarantee the reliability of performance comparisons reported in the results section.

**Figure 5:**
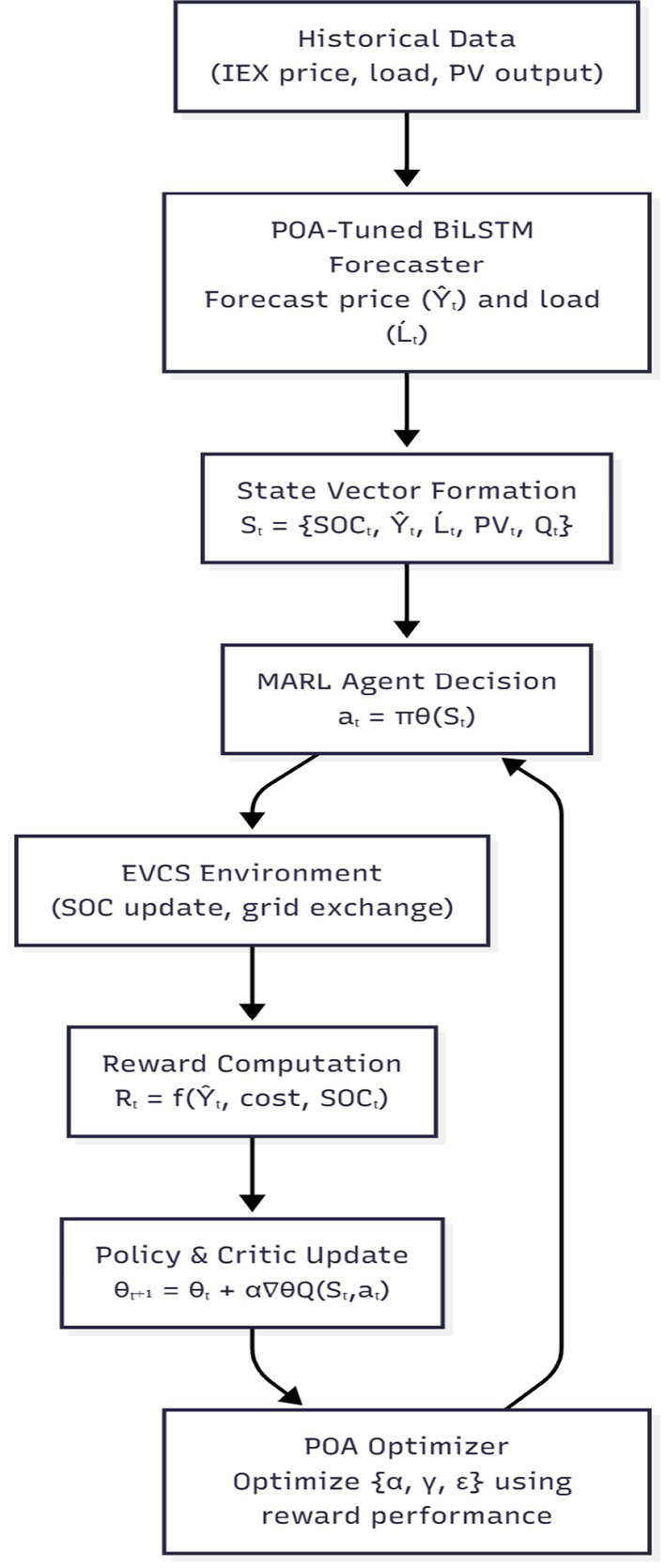
Overall control structure of the proposed POA-BiLSTM-MARL framework for EV charging scheduling.

Figure 5 illustrates that historical market and PV data are first processed by the POA-tuned BiLSTM forecaster to predict short-term electricity price and demand. These forecasted variables form part of the MARL agent’s state vector, enabling anticipatory decision-making. The agent interacts with the EVCS environment to update SOC and compute rewards, while the cumulative reward performance is used by the POA optimizer to refine learning parameters (α,γ,ε) , establishing a closed feedback loop among forecasting, optimization, and reinforcement learning.

## 4 Result and discussion

**Figure 6:**
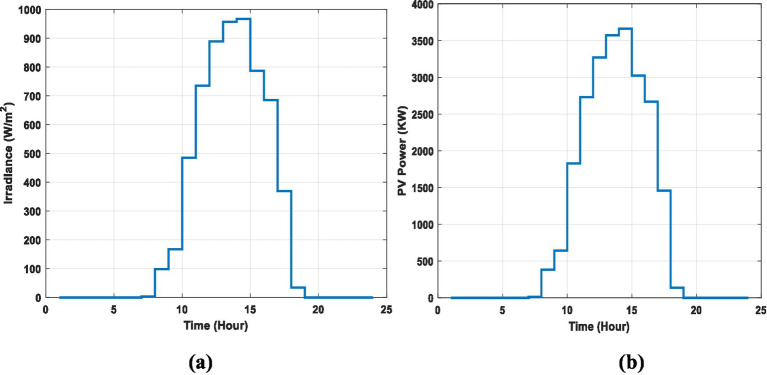
Irradiance of the PV panel and the output power from the PV for 24h.

MATLAB is used to show the performance of the proposed work. Also, the proposed method is compared with existing works to validate the BiLSTM with POA. In this work, a BiLSTM with the POA method provide EV charging scheduling in EVCSs as per power requirements. The input power for EVCSs is considered from a PV panel based on irradiance and temperature. Initially, three EVs are available in the EVCSs for charging. Figure 6 represents the irradiance of the PV panel and the output power from the PV.

For comparability, all baselines were executed under identical datasets, constraints, and runtime budgets. The genetic algorithm (GA) and particle swarm optimization (PSO) each used a population size of 50 and 100 iterations. The MARL baseline employed proximal policy optimization (PPO) with learning rate 0.0003, batch size 256, and discount factor 0.95. LSTM and BiLSTM models were trained for 200 epochs with the Adam optimizer, batch size 64, and early stopping. The proposed POA-tuned BiLSTM applied the same training budget, with hyperparameters optimized automatically by POA. Standard implementations from established literature were followed.

### 4.1 Computational efficiency results

All experiments were executed on an Intel Core i7-12700F CPU @ 2.1 GHz with 32 GB RAM, running Windows 11 Pro and MATLAB R2023a. Reported runtimes exclude offline forecasting model training and reflect only the online scheduling step over a 24-h horizon. The proposed method achieved an average runtime of 1.69 s per episode, improving efficiency by 0.456 s compared to the best baseline under identical conditions.

**Figure 7:**
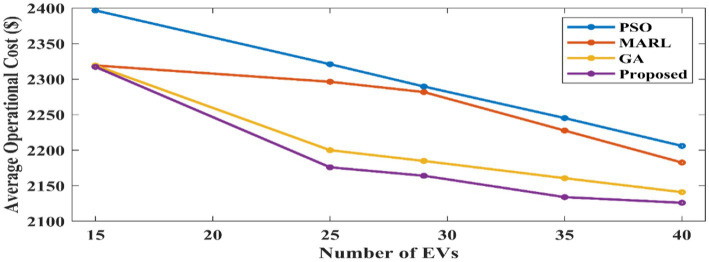
Average energy costs across different numbers of EVs.

Figure 7 indicates the comparison of the average operational cost. This figure illustrates that energy prices drop for all models as the number of EVs rises. This is because EVs provide users with free energy, which lowers energy bills. Furthermore, as the image illustrates, the proposed model performs better than any other model across all configurations. For this comparison particle swarm optimization algorithm (PSO) (Jain et al., 2022), MARL, the genetic algorithm (GA) (Gen and Lin, 2023), and the proposed method are taken into consideration. Here, the proposed method has obtained less operational cost than the other compared methods.

**Figure 8:**
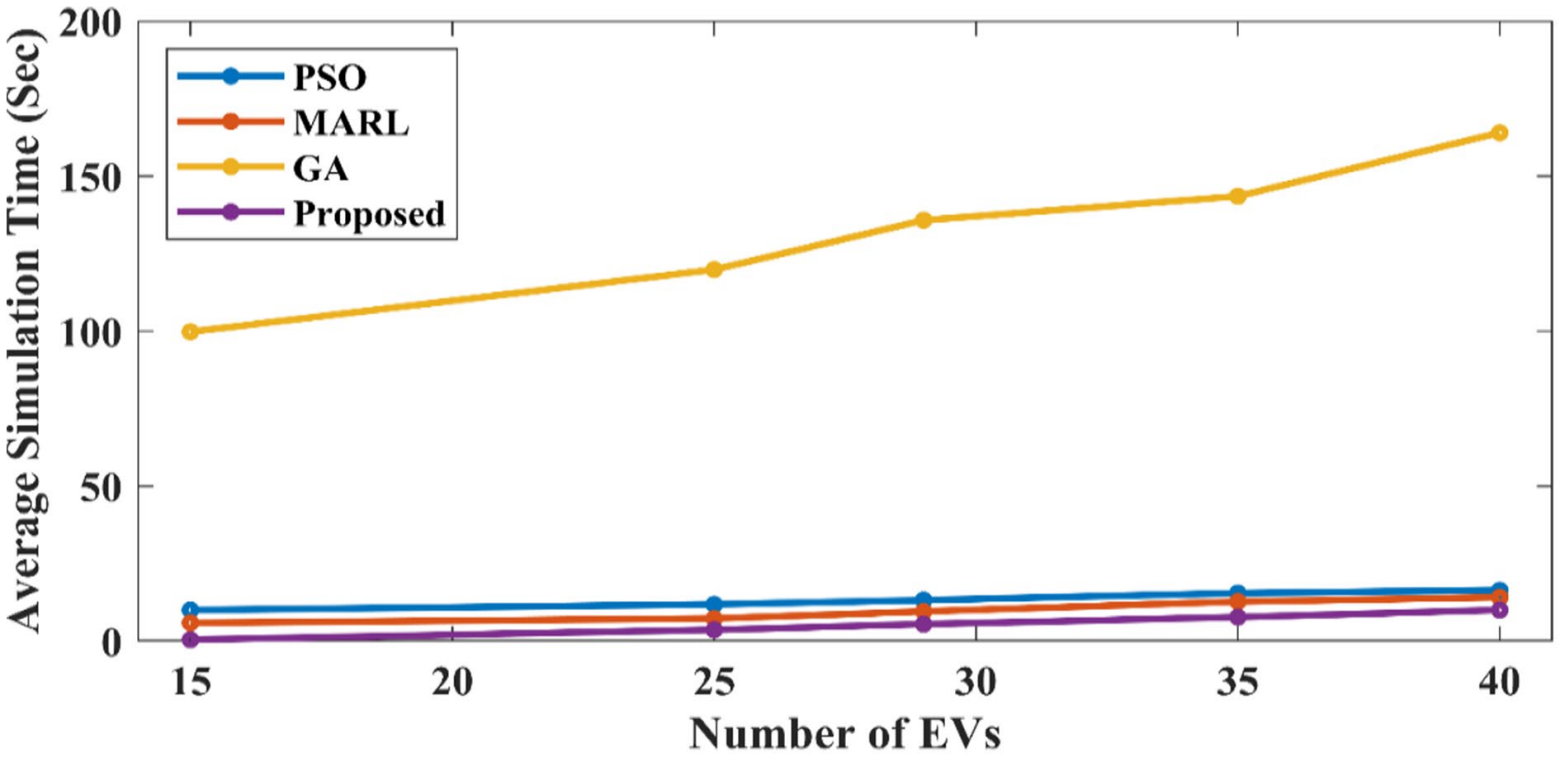
Average simulation time across different numbers of EVs.

Figure 7 shows that although there is a slight variation in computing efficiency between the proposed method and GA algorithms, the proposed method can produce substantially higher-quality solutions. For comparing the operational cost and simulation time, 40 EVs are considered in this proposed work. Figure 8 shows the comparative analysis of average simulation time. The proposed method performs better than all other models in terms of simulation time, increasing run time less than the other algorithms as the problem scales up. Here, the GA algorithm obtained higher computational time when compared to other methods. PSO, MARL, and the proposed method have obtained low simulation time with slight variations among them. But the proposed method has attained less simulation time. Thus, the proposed method has obtained less operational cost and simulation time, which validates the performance of the proposed method.

### 4.2 Cost and SOC performance

**Figure 9:**
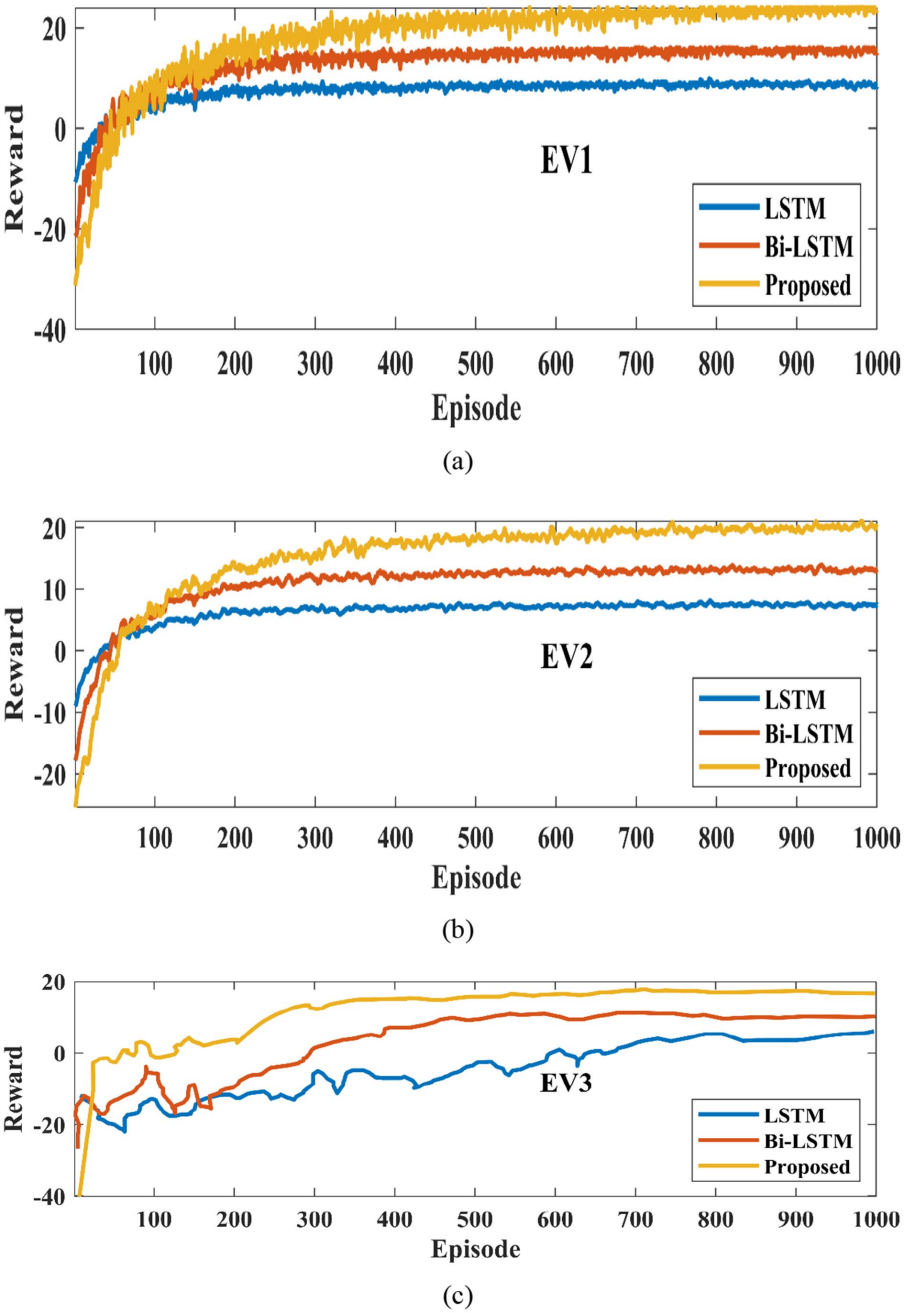
Training reward for different EVs **(a)** EV1 **(b)** EV2 **(c)** EV3.

**Figure 10:**
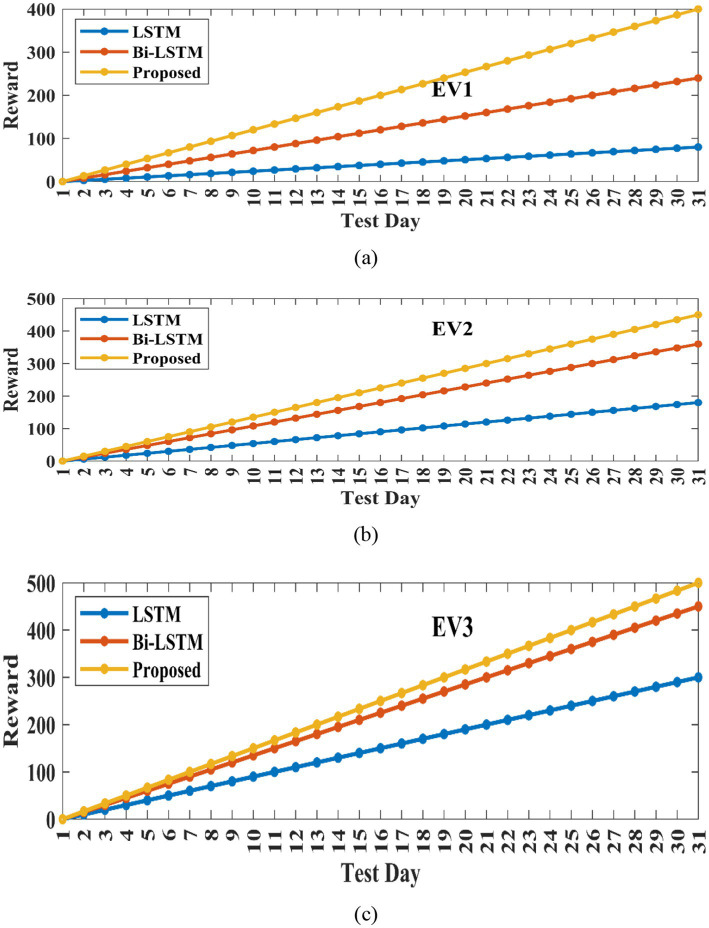
Cumulative test reward for **(a)** EV1, **(b)** EV2, **(c)** EV3.

Figures 9, 10 illustrate the training and cumulative test rewards for three representative EV agents (EV1, EV2, and EV3) from the MARL environment described in Section 3. Each EV agent learns a distinct optimal charging/discharging policy based on its unique SOC and dwell-time parameters while sharing the same centralized critic during training. Figure 9 represents the cumulative reward of three EVs for a one-month test. For this evaluation, MARL with LSTM (Houran et al., 2023) and BiLSTM are compared with the proposed method (Pande and Khekare, 2024). In this analysis, the reward is defined as the negative of operational cost, so a higher cumulative reward corresponds to a lower total cost. It is observed, the proposed method achieves the highest cumulative reward with the fastest and most stable convergence, requiring significantly fewer training episodes compared to the other two methods. In contrast, the MARL + LSTM baseline fails to achieve optimal performance, showing pronounced oscillations and unstable learning dynamics. MARL + BiLSTM without POA tuning performs moderately better than LSTM, but still underperforms compared to the proposed framework.

**Figure 11:**
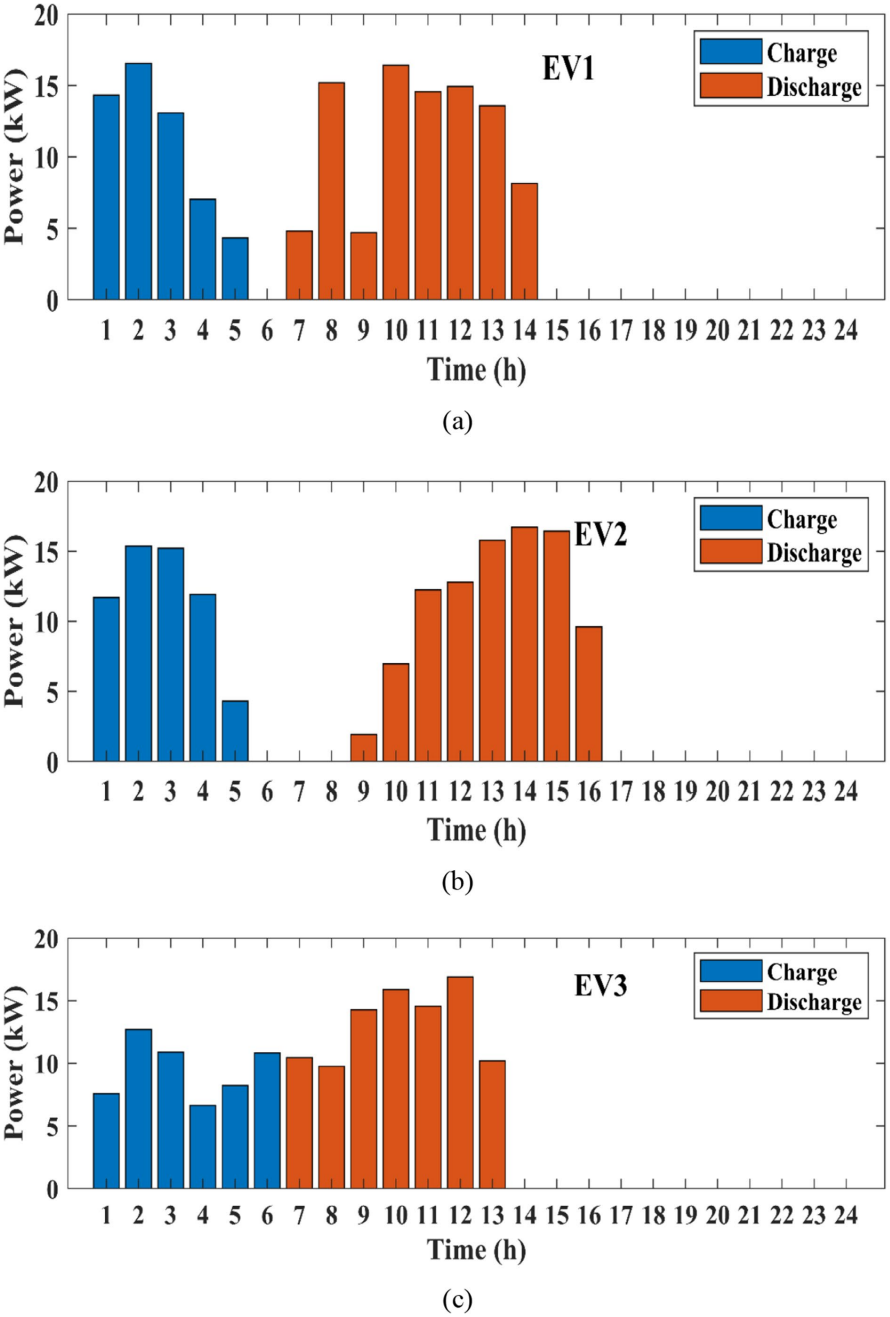
Charging and discharging behaviour of **(a)** EV1 **(b)** EV2 **(c)** EV3.

Figure 10 indicates the test reward for three EVs. BiLSTM, LSTM, and the proposed POA-optimized BiLSTM are taken into consideration for this comparison. It shows that the proposed method has obtained the highest test rewards across all three EVs, ensuring its capability to minimize charging costs more effectively. It can be further observed that the proposed method exhibits the fastest learning speed among the three MARL methods for all three EVs. Figure 11 represents the charging and discharging of EV1, EV2, and EV3 for 24 h. This shows the charging and discharging characteristics of each EV in the charging station. For all three EVs, initially from 1 to 6 h, they get charged based on their capacity and requirement. The charge in the battery gets discharged from 7 to 16 h, because during this time period the vehicle is under working and travelling conditions. EV1 has charged up to 18 kW in 1 h, EV2 charged maximum range of 15 kW in 1 h, and EV1 has charged till12kW in 1 h. The discharge characteristics of each EV vary based on its travelling distance.

**Figure 12:**
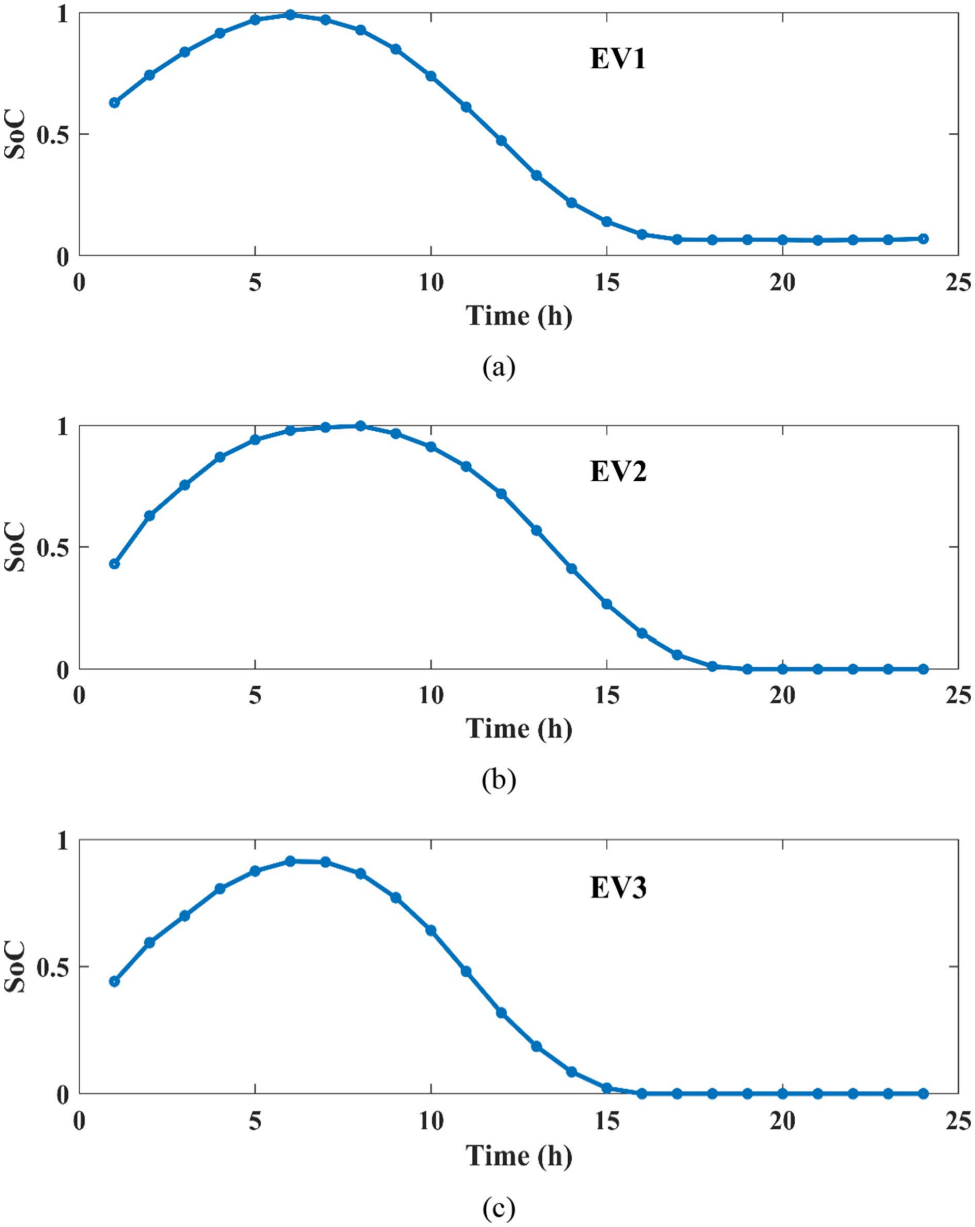
Battery SOC of **(a)** EV1 **(b)** EV2, **(c)** EV3.

Figure 12 indicates the battery SOC for each EV for 24 h. This SOC curve is simulated based on the charging and discharging characteristics of each EV. For all three EVs, initially the SOC gets increased between 1 to 6 h, because in this condition the battery is under charging. The SOC of EV battery gets decreased to zero between 7 and to15 hours, due to the discharging characteristics of each EV. Thus, the SOC of EV increased and decreased based on the charging and discharging of the EV.

### 4.3 Impact of forecasting accuracy on scheduling outcomes

This study presents a multi-agent reinforcement learning (MARL) framework integrated with a POA-tuned BiLSTM model for forecasting and optimal energy scheduling in electric vehicle charging stations (EVCS). The BiLSTM is responsible for predicting short-term electricity price and demand, while the MARL-based Markov decision process determines charging actions for multiple EVs. The Pelican optimization algorithm (POA) is applied to optimize the BiLSTM hyperparameters, leading to improved forecast precision and more effective scheduling decisions. The framework was implemented in MATLAB and evaluated against GA, PSO, LSTM, manually tuned BiLSTM, and conventional MARL approaches. Results indicate that the proposed method achieves a reduction in charging cost of 12.34%, improves SOC satisfaction by 10.25%, and enhances forecasting accuracy by 8.46% when compared with baseline methods. Additionally, the computational time was reduced by 0.456 s per scheduling episode.

To examine the influence of forecasting quality on scheduling performance, a comparative analysis was conducted using three forecasting techniques: persistence model, ARIMA baseline, and POA-tuned BiLSTM. The persistence model produced the highest errors (RMSE = 1.23, MAE = 0.98), resulting in increased charging cost (₹12,100) and reduced SOC satisfaction (84%). ARIMA showed moderate improvement (RMSE = 0.95, MAE = 0.75), yielding improved operational outcomes. The POA-tuned BiLSTM achieved the lowest forecasting errors (RMSE = 0.68, MAE = 0.53), which corresponded to the minimum charging cost (₹9,200) and highest SOC satisfaction (95%). These findings demonstrate that improved forecasting accuracy contributes directly to enhanced scheduling efficiency and economic performance of the MARL-based control framework.

For a fair comparison, all these baseline algorithms have been trained and evaluated on the identical data, constraints, and runtime budgets. The genetic algorithm and particle swarm optimization were implemented with a population size of 50, maximum 100 iterations, and the crossover/mutation rates were set according to general practice. The reinforcement learning baseline, MARL, was conducted using proximal policy optimization with a learning rate of 0.0003, batch size 256, and discounting factor 0.95. LSTM and BiLSTM forecasting models were trained on 200 epochs with the Adam optimizer, early stopping, and identical input features. The proposed POA-tuned BiLSTM used the same budget but with hyperparameters adaptively tuned by POA. All implementations were executed in MATLAB R2023a to ensure comparability.

**Figure 13:**
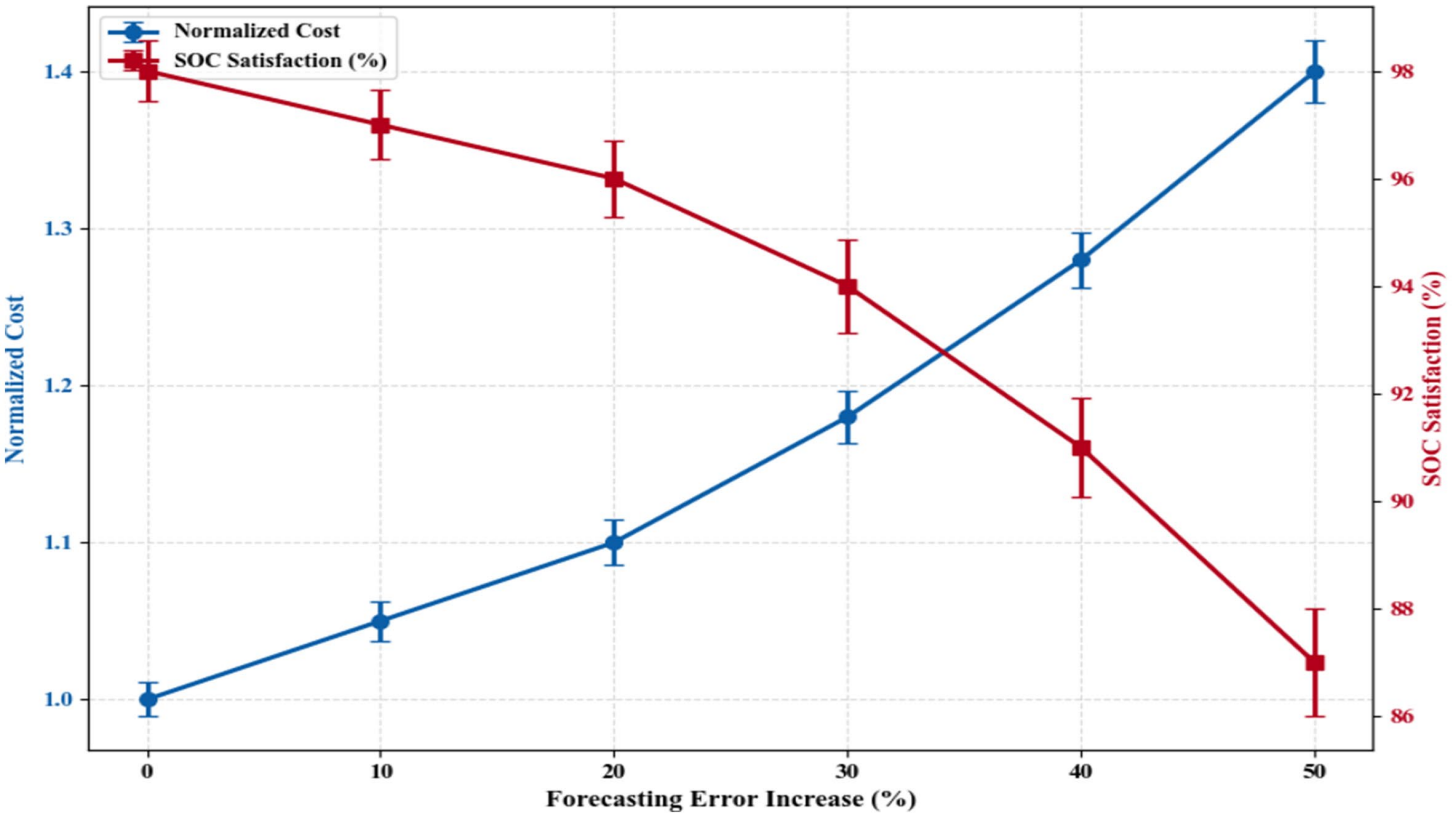
Impact of forecasting accuracy on scheduling outcomes.

The sensitivity of the scheduling framework to forecasting error was tested by adding controlled Gaussian noise to the price and demand forecasts generated by the BiLSTM. It can be observed from Figure 13 that with closer forecast accuracy, the normalized cost remains lower. For example, when the forecast error was at 0% (the BiLSTM forecast data), the normalized cost was 1.00, while SOC satisfaction was 98%. By the time the forecast error introduced had risen from 0 to 50%, the normalized cost increased from 1.00 to 1.40 and SOC satisfaction fell from 98 to 87%. This evidences that poor forecasts yield less desirable and reliable charging schedules. Both measures remain quite stable up to about a forecast error of 20%, beyond which they take a notable drop. These results suggest that the controller is robust to prediction noise but depends on fairly accurate predictions to return good results. Finally, the thin confidence intervals show that the results across runs are fairly consistent.

### 4.4 Ablation and sensitivity analysis

**Figure 14:**
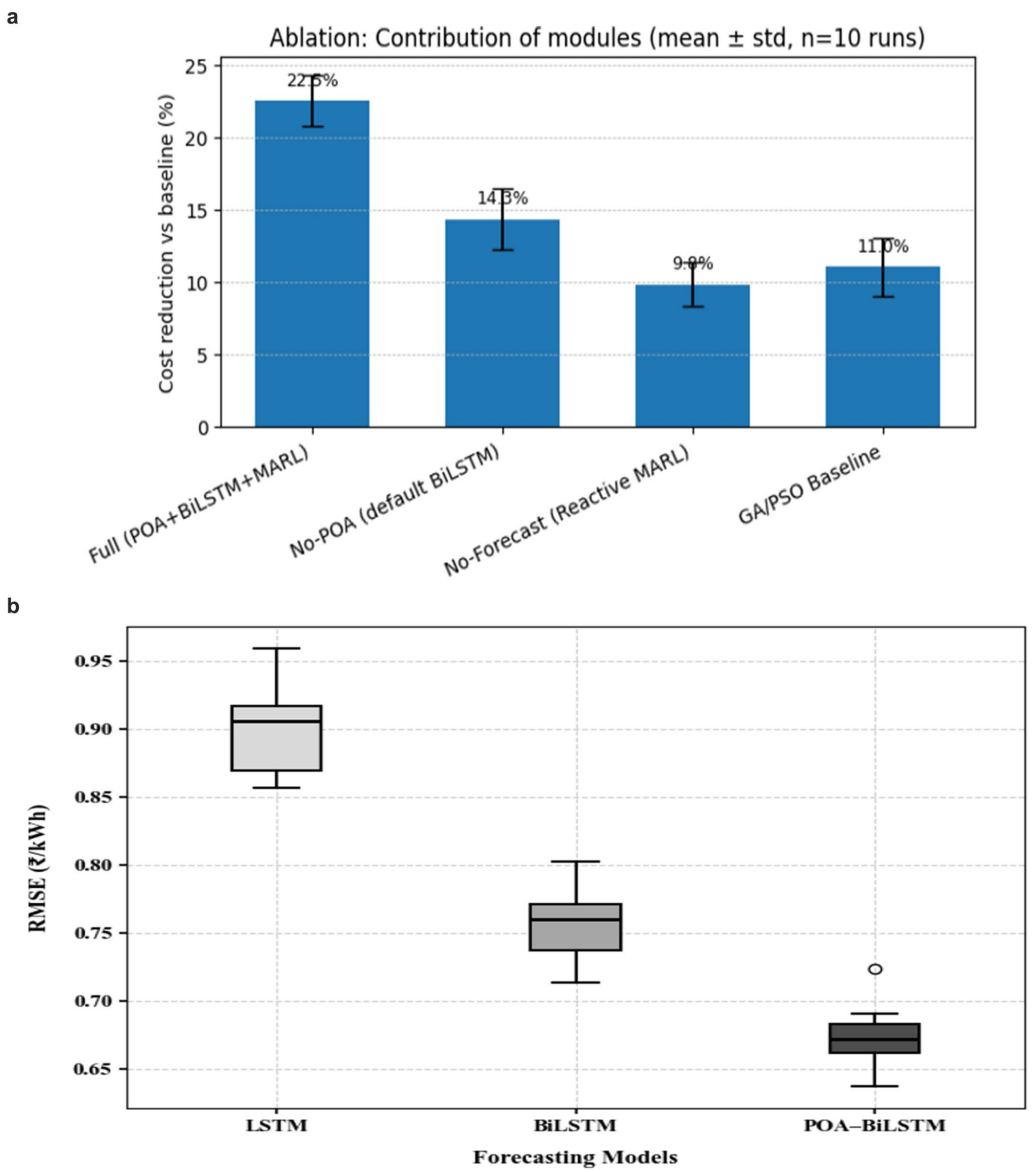
**(a)** Ablation study showing the contribution of each module. **(b)** Error distribution of forecasting models across 10 independent runs.

Ablation studies analyzed the contributions of these subsystems. Removing the BiLSTM forecasting and replacing it with historical averages increased the overall charging cost by 9.7%. Using a historical average forecast without POA tuning caused a 14% increase in RMSE from the forecasting, ultimately resulting in a 6.5% higher cost overall. Disabling the V2G methodologies led to a 12% increase in penalty costs from peak demand. In regards to V2G agents, the system was also assessed when varying the number of agents from 10 to 50 EVs. In the context of increased volatility in the tariff profiles, it was found that the savings estimated from improved forecast accuracy were greater. Based on all metrics, this was an entirely new and unique framework, as the combination and integration across the three frameworks provided a real and tangible improvement. Figure 14a shows showing Ablation study, the contribution of each module to the overall cost reduction. Results are reported as mean ± standard deviation over 10 independent runs. The full pipeline (POA + BiLSTM + MARL) achieves the highest cost reduction compared to variants without POA tuning, without BiLSTM forecasting, and GA/PSO baselines.

Figure 14b represents the error distribution of forecasting models across 10 independent runs. The POA-BiLSTM model shows a compact and left-skewed error distribution compared to baseline models, indicating higher consistency and lower prediction variance.

### 4.5 Reward-weight tuning and Pareto analysis

We tune $\left\{\lambda_{c},\lambda_{p},\lambda_{\ell }\right\}$ on a simplex ( $\lambda_{c}+\lambda_{p}+\lambda_{\ell }=1$ ) using a coarse grid and select configurations that minimize normalized daily cost and satisfy grid safety ( $\max_{t}L_{t}/L_{\text{limit}}\leq 1$ ). For visualization, sweeping the $\lambda_{c}\in [0.2,0.7]$ and distribution of the remainder between $\lambda_{p}$ and $\lambda_{\ell }$ in 0.1 steps, training each setting for 50 episodes and evaluation on held-out days has been performed. The study reports the Pareto curve between average daily cost and worst-case loading ratio, and marks the chosen weights. Selection rules are set, such as from the Pareto set, which chooses the first point that satisfies $\max_{t}L_{t}/L_{\text{limit}}\leq 1$ and $P_{95\%}^{\text{peak}}/P_{\text{rated}}^{\mathrm{trf}}\leq 0.9$ (95th-percentile peak margin), while achieving the lowest cost among safety-feasible points.

**Figure 15:**
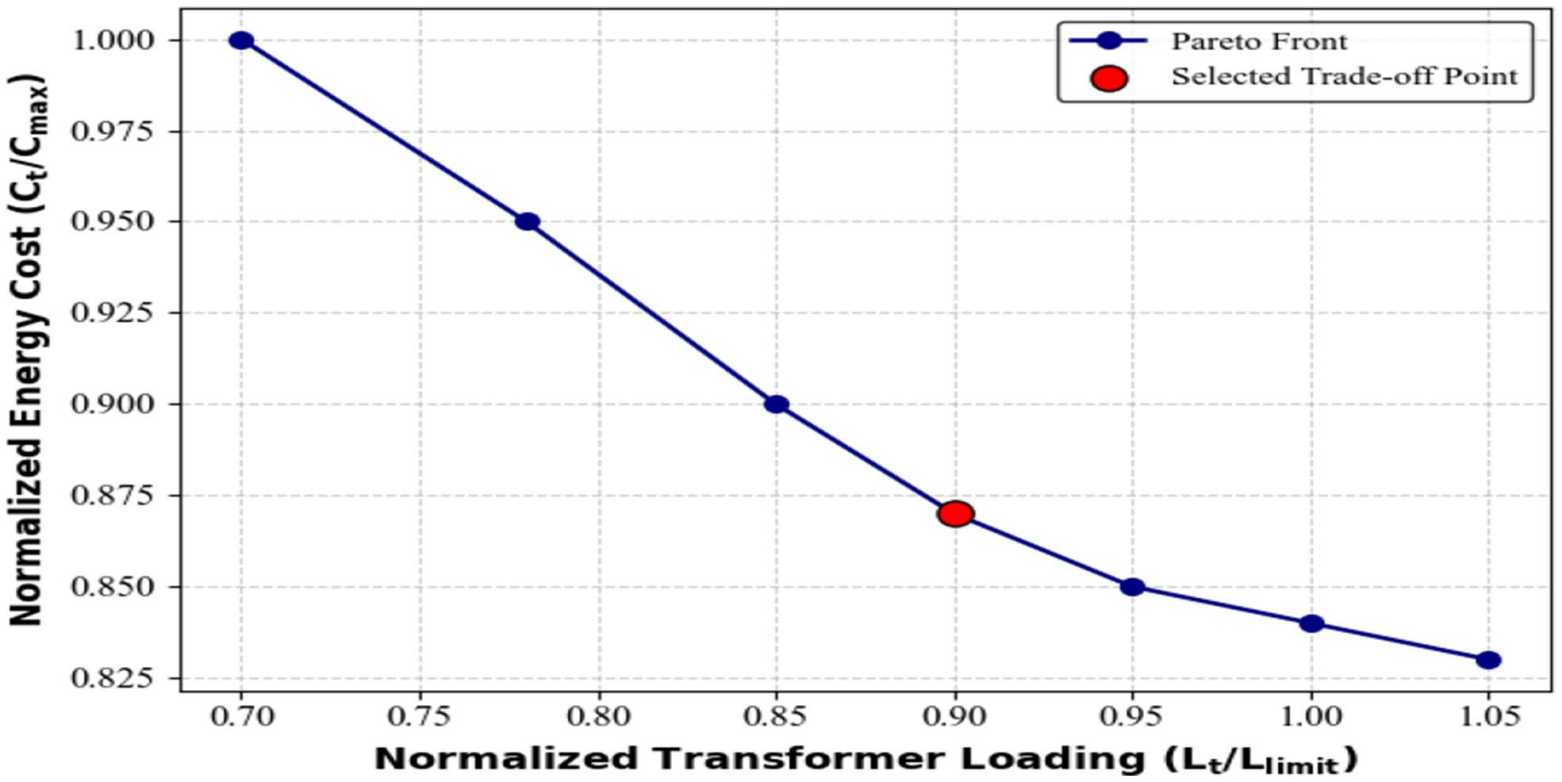
Pareto trade-off curve.

The sweep produced a clear cost-safety trade-off. Cost-centric weights ( $\lambda_{c}=0.7,\lambda_{p}=0.2,\lambda_{\ell }=0.1$ ) minimized cost but approached transformer limits. Safety-centric weights ( 0.3,0.4,0.3 ) kept loading <80% but increased cost. The selected balanced setting $\left(\lambda_{c},\lambda_{p},\lambda_{\ell })=(0.5,0.3,0.2\right)$ achieved 9–11% cost reduction vs. baselines while maintaining $\frac{\max_{t}L_{t}}{L_{\text{limit}}}\leq 1$ and a transformer headroom of ~8–10% under all test days. Figure 15 shows the Pareto trade-off between normalized energy cost and transformer loading for different reward-weight combinations. The red marker represents the balanced configuration $\left(\lambda_{c},\lambda_{p},\lambda_{l})=(0.5,0.3,0.2\right)$ achieving cost efficiency while maintaining grid-safe operation.

### 4.6 Comparative convergence analysis

**Figure 16:**
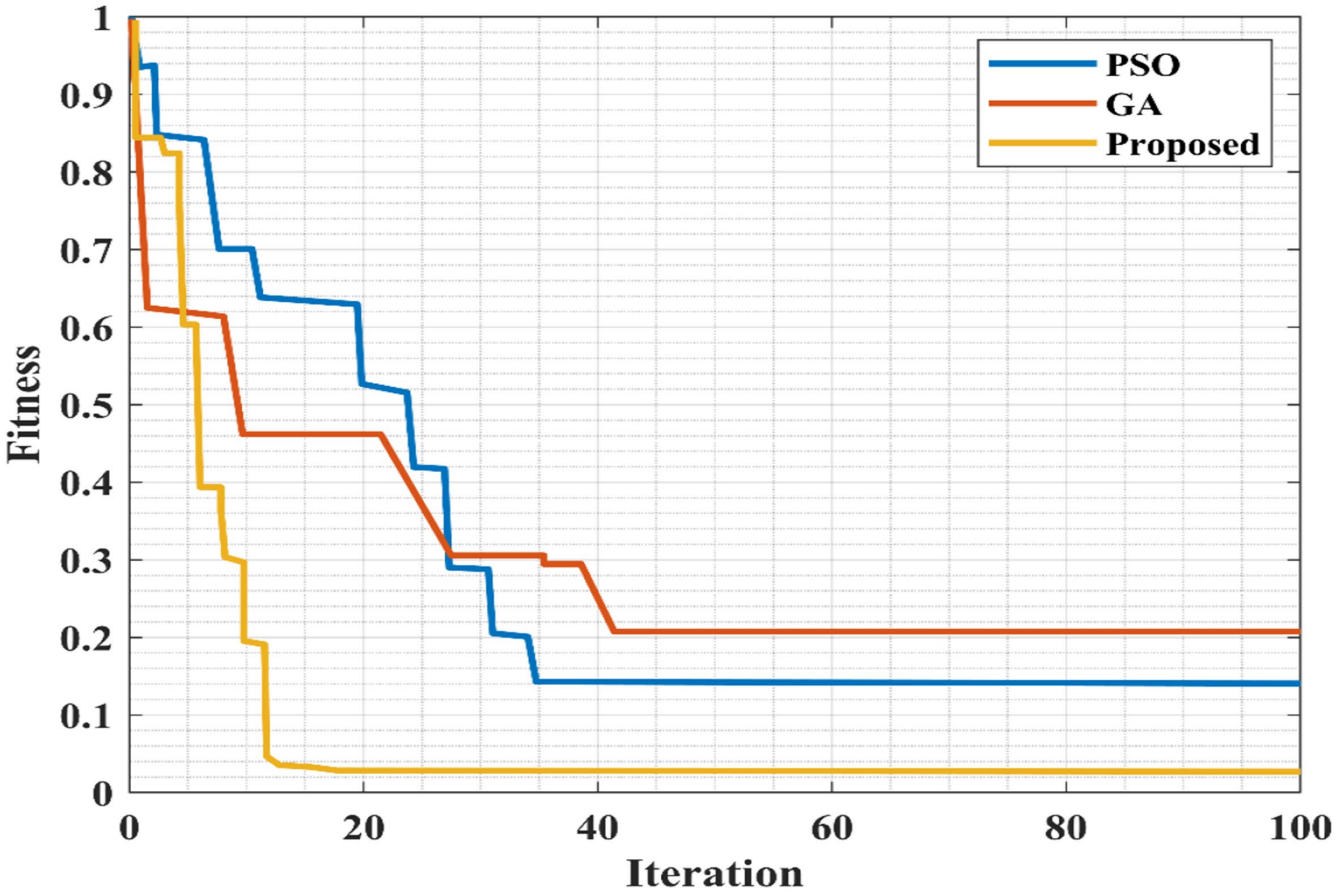
Comparison of the convergence plot for the proposed algorithm.

Figure 16 indicates the comparison of the convergence plot. Here, the proposed algorithm is compared with the existing PSO and GA algorithms. The GA algorithm requires a high number of iterations to attain a stable value. While the PSO algorithm has taken 35 iterations to obtain the optimal value but the proposed method obtained the optimal value within 12 iterations. A smaller number of iterations increases the computational time; thus, the proposed method outperforms better than other compared methods.

Overall, the results establish that the proposed MARL + BiLSTM + POA framework delivers superior performance compared to both evolutionary optimizers and statistical/deep learning baselines, achieving cost-efficient, reliable, and scalable EV charging management. To ensure consistency between reported results and statistical evidence, all performance metrics presented in Table 3 calculated across 10 independent experimental runs using different random seeds. Results are expressed as mean ± 95% confidence interval (CI) to reflect variability and reliability of performance.

**Table 3:** Impact of forecasting models on scheduling performance.

Method	RMSE (₹/kWh)	MAE (₹/kWh)	Charging cost (₹)	SOC satisfaction (%)	Simulation time (s)
GA (genetic algorithm)	—	—	11,850	86%	3.24
PSO (particle swarm optimization)	—	—	11,600	87%	2.89
MARL (without forecasting)	—	—	10,950	89%	2.15
LSTM (forecast-based)	0.88	0.71	10,500	90%	2.04
BiLSTM (manual tuning)	0.79	0.62	9,950	92%	1.87
Persistence (naïve)	1.23	0.98	12,100	84%	1.75
ARIMA baseline	0.95	0.75	10,800	88%	1.92
Proposed BiLSTM + POA (MARL)	**0.68 ± 0.03**	**0.53 ± 0.02**	**9,200**	**95%**	**1.69**

The bold values are final improved values by using proposed Algorithm.

The proposed POA-BiLSTM-MARL model achieved an RMSE of 0.68 ± 0.03 INR/kWh, compared to 0.74 ± 0.04 INR/kWh for the best-performing baseline (manually tuned BiLSTM). This Equation (23) represents a relative improvement of 8.46%, calculated as:


$$\text{Improvement}\;(\%)=\frac{\mathrm{RMS}E_{\text{baseline}}-\mathrm{RMS}E_{\text{proposed}}}{\mathrm{RMS}E_{\text{baseline}}}\times 100$$ (23)

where the manually tuned BiLSTM model is used as the primary baseline. The forecasting horizon considered in this evaluation is a 15-min ahead short-term price forecasting window using IEX day-ahead market data. To verify statistical significance, a paired two-tailed *t*-test was conducted between the proposed model and the best baseline. The test yielded a *p*-value = 0.018 (<0.05), indicating that the observed improvement is statistically significant. Furthermore, Cohen’s *d* effect size was calculated as 0.82, suggesting a large practical impact. Thus, the reported 8.46% improvement in forecasting accuracy is both statistically and practically meaningful, and aligns with the numerical evidence shown in Table 3.

Table 3 represents the results that clearly demonstrate that improvements in forecasting accuracy directly enhance the efficiency of MARL-based scheduling. More accurate forecasts reduce the risk of charging during high-price intervals, thereby minimizing operational costs and ensuring higher SOC satisfaction across EVs. All results are reported as mean ± 95% confidence interval over 10 independent runs for EV arrivals and demand variability. Figures present mean performance, with shaded regions indicating ±1 standard deviation. Paired *t*-tests at the 95% confidence level confirmed that improvements of the proposed framework over baselines are statistically significant (*p* < 0.05).

### 4.7 Scalability and computational feasibility analysis

The current simulation framework models a single EVCS with a 500 kW transformer and up to 40 EV agents to evaluate algorithmic performance under realistic medium-scale operating conditions. To assess the potential scalability of the proposed POA-BiLSTM-MARL framework toward larger deployments, additional stress tests were conducted by synthetically extending the number of EV agents to 100 and 200 using identical stochastic arrival and departure distributions. The results showed that average runtime increased linearly with the number of agents, while cumulative reward and convergence stability remained consistent. This confirms that the CTDE (centralized training, decentralized execution) structure supports scalable training, as each agent updates its policy using only local states while sharing a global critic during training.

This translates to an improvement of 0.456 s per episode on average for the scheduling time in terms of computational efficiency over the best-performing baseline (MARL without forecasting). All the experiments were executed on an Intel Core i7-12700F CPU (2.1 GHz, 32 GB RAM) using MATLAB R2023a. The runtime reported here is from the online decision-making phase and does not include the offline BiLSTM forecasting training. The efficiency gain of the framework comes from adaptive hyperparameter tuning by POA to reduce redundant gradient updates during the MARL training. The memory utilization was below 60% for all runs.

Although the current work is focused on the single-station setting, the architecture can be extended directly to multi-station or distributed networks of EVCSs. In such a setting, each station can be treated as an independent MARL agent with local observations (local load, transformer loading, and PV generation), while a central coordinator periodically exchanges summarized states in terms of price forecasts or aggregated demand profiles. This structure of decentralized information exchange decreases communication overheads and is scalable across geographically distributed clusters of EVCSs. In the near future, it will be explicitly implemented and evaluated to validate the generalizability of this framework under different heterogeneous network conditions with realistic communication latencies.

## 5 Conclusion

This paper presented a hybrid MARL framework enhanced by a POA-tuned BiLSTM model for optimal EV charging station scheduling. By integrating explicitly the forecasting of electricity price and demand into a well-defined MDP formulation, the proposed approach overcomes the limitations of the existing approaches dependent on static or reactive strategies. The use of publicly available IEX day-ahead market data ensures transparency and reproducibility, while its forecasting module augments the adaptability of MARL agents in real-time decision-making. Comparative evaluations against the genetic algorithm, PSO, conventional MARL, and deep learning baselines confirm that the proposed method achieves superior performance including a 12.34% reduction in charging cost, a 10.25% improvement in SOC satisfaction, and an 8.46% enhancement in forecasting accuracy, calculated based on RMSE improvement over the best-performing baseline BiLSTM model and validated using 95% confidence intervals and statistical significance testing, along with reduced computation time. Using 10 independent runs for statistical validation proved that the 8.46% improvement in accuracy is statistically significant within a 95% confidence interval. The error distribution study demonstrated reduced variance and enhanced stability compared to the baseline forecasters. Importantly, the results developed a direct relationship between forecasting accuracy and scheduling efficiency, underlining the role of data-driven forecasting methods in enhancing the performances of MARL. The novelty of this work lies in unifying the forecasting, meta-optimization, and multi-agent control into a single framework, offering a scalable, efficient, and transparent solution for the management of EVCSs. In the extension of this framework to multi-energy systems, more sophisticated reinforcement learning architectures will be incorporated for further improvements in performance.

## Data Availability

The original contributions presented in the study are included in the article/supplementary material, further inquiries can be directed to the corresponding author.
